# Indoor Pedestrian Self-Positioning Based on Image Acoustic Source Impulse Using a Sensor-Rich Smartphone

**DOI:** 10.3390/s18124143

**Published:** 2018-11-26

**Authors:** Xiyu Song, Mei Wang, Hongbing Qiu, Liyan Luo

**Affiliations:** 1 Ministry of Education Key Laboratory of Cognitive Radio and Information Processing, GuiLin University of Electronic Technology, Guilin 541004, China; songxiyu@guet.edu.cn; 2 College of Information Science and Engineering, Guilin University of Technology, Guilin 541004, China; mwang@guet.edu.cn; 3 Wireless Broadband and Signal Processing Guangxi Key Laboratory, Guilin University of Electronic Technology, Guilin 541004, China; xiaoyan12027@gmail.com

**Keywords:** indoor acoustic localization, acoustic image model, motion dynamics information, pedestrian dead reckoning, smartphone-based self-positioning

## Abstract

The ubiquity of sensor-rich smartphones provides opportunities for a low-cost method to track indoor pedestrians. In this situation, pedestrian dead reckoning (PDR) is a widely used technology; however, its cumulative error seriously affects its accuracy. This paper presents a method of combining infrastructure-free indoor acoustic self-positioning with PDR self-positioning, which verifies the rationality of PDR results through the acoustic constraint between a sound source and its image sources. We further determine the first-order echo delay measurements, thus obtaining the mobile user position. We verify that the proposed method can achieve a continuous self-positioning median error of 0.19 m, and the error probability below 0.12 m is 54.46%, which indicates its ability to eliminate PDR error, as well as its adaptability to environmental disturbances.

## 1. Introduction

The increasing number of sensor-rich smartphones has raised interest in using their sensors for indoor localization applications, such as indoor navigation [1], location-based services [2], providing aid for hearing-impaired persons [3], and environmental perception [4,5]. Global positioning system (GPS) can provide effective localization results for pedestrians in outdoor environments, but may not be useful for indoor environments due to weak signal reception and the indoor shadowing effect [6]. Therefore, indoor pedestrian self-positioning technology has attracted considerable attention.

Based on specific technology, it is possible to categorize methodologies for smartphone-based indoor pedestrian self-positioning systems into two distinct groups: (1) infrastructure-based systems that use auxiliary equipment or a cooperation between nodes to realize target tracking [1,2,3,4,5,6,7,8,9], and (2) the infrastructure-free systems that realize pedestrian self-positioning using only the information provided by the smartphone carried on one’s person [9,10,11,12,13,14,15,16,17,18,19,20]. However, when using the former, the pedestrian is likely to experience difficulties in position acquisition when the cooperative information is unavailable. The widely used pedestrian dead reckoning (PDR) method only provides a relative position estimate, with its accuracy degrading over time. The fusion of other positioning methods has been proposed to solve this problem [21,22,23,24,25,26]. Yang et al. [23] proposed a novel smartphone-based indoor localization system that improved the PDR results by integrating an infrastructure-based acoustic localization system, reaching sub-meter localization accuracy at the expense of a complicated data availability analysis and computational complexity.

To reduce the influences of noise on the source tracking, the motion and observation models of the moving source as well as the probability distribution model of the errors [27] must be established for filtering methods, i.e., Kalman filter, particle filter, and their variants [26]. Such complications and inconveniences limit the applications of the filtering methods.

To alleviate the aforementioned problems, this study provides an acoustic constraint algorithm to verify the rationality of the PDR results, which reduces the cumulative errors by using the geometric relationship between the sound source and its image sources.

The rest of this paper is organized as follows. Section 2 provides an overview of the proposed indoor pedestrian self-positioning system. Section 3 details the first-order echo estimates based on an acoustic image model (AIM). In Section 4, we describe the solution for the first-order echo measurements in three steps: the calculation of the cross-correlation, the calculation of first-order echo measurements; and the acoustic principle-based constraints. Section 5 summarizes the applied Levenberg–Marquardt algorithm-based weighted nonlinear least squares (LMA-WNLS) model for pedestrian position values. Section 6 highlights the performance of the proposed method and the results analysis, which proves the effectiveness of the proposed system for indoor pedestrian continuous position acquisition. The conclusions are drawn in Section 7.

## 2. System Overview

**Figure 1:**
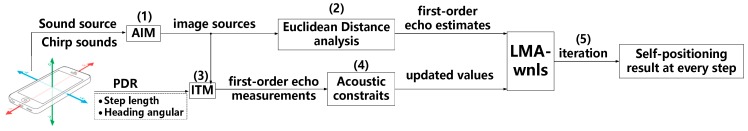
Overview of the proposed system architecture. AIM: Acoustic Image Model. ITM: Isosceles Trapezoid Model; LMA-WNLS: Levenberg–Marquardt algorithm-based weighted nonlinear least squares.

We assume that a sounding smartphone is always carried by the indoor pedestrian. The pedestrian moves autonomously inside a room. At every step, the loudspeaker of the smartphone produces a chirp pulse, the microphone of this smartphone registers the echoes, and the inertial sensors record the accelerometer and gyroscope readings. We define the room to be a K-faced rectangular room, which is widely used in teaching buildings. The pedestrian is modeled in a room as a point source in a rectangular cavity, and thus, for ease of explanation, the pedestrian and the sound source (the loudspeaker of the user smartphone) are hereafter equivalently used in this paper. We worked in two-dimensional (2D) space, ignoring the floor and the ceiling, given K=4, but the results could be extended to three-dimensional space. The proposed system was implemented to achieve submeter-level positioning accuracy and reliability. To this end, five steps were followed to obtain the position of the indoor pedestrian, as presented in Figure 1.

**Figure 2:**
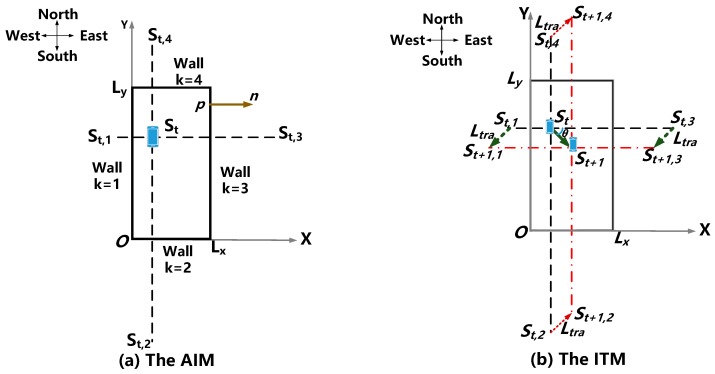
Illustration of the spatial geometrical models presented in this paper. We suppose the room size is $\left[L_{x},L_{y}\right]$, and the origin point is located at O(0,0). (**a**) The acoustic image model (AIM) for the first-order images. Point p is an arbitrary point of the kth wall. Vector n is the outward-pointing unit normal associated with the kth wall, $S_{t,k},\;k=1,\ldots ,K$ are the first-order image sources of $S_{t}$ corresponding to the kth wall. (**b**) The isosceles trapezoid model (ITM) for a moving sound source. When the sound source moves from $S_{t}$ to $S_{t+1}$, $S_{t,k}$ moves to the $S_{t+1,k}$, k = 1, …, 4, respectively; then, these points ($S_{t},S_{t,k},S_{t+1},S_{t+1,k}$) can form a set of isosceles trapezoids with the waist length represented as the step length $L_{tra}$ and the inner angle as the heading angular θ. The step forward from $S_{t}$ to $S_{t+1}$ is shown as a green full line, the sound rays at time t are the blue dashed line, and sound rays at time t+1 are the red dotted line.

The first step is to compute the image sound sources, denoted as $S_{t,k},\;k=1,\ldots ,4$ as shown in Figure 2, without loss of generality. One corner marked with O in the room is placed as the origin based on the AIM [28,29]. Then, Euclidean distance analysis is applied for the first-order echo estimates, which are detailed in Section 3. An isosceles trapezoid geometry [20] was adopted to calculate the first-order echo measurements based on the PDR information (i.e., the step length $L_{tra}$ [30] and the heading angular θ [31]) and the locations of all $S_{t,k}$. The fourth step is to exploit the acoustic constraits to update the measurementsvalues. Lastly, the LMA-WNLS is performed, which is used to quickly iterate the current pedestrian position coordinates and achieve the tracking effect. The LMA-WNLS is detailed by Mensing [32], and a brief summary is provided in Section 5.

## 3. First-Order Echo Estimates

In the AIM, the reflections from the walls are replaced with signals produced by image sound sources across the corresponding walls. For a first-order echo and the kth wall described by the outward-pointing unit normal $n_{k}$ and an arbitrary wall point $p_{k}$, the image sources $S_{t,k}$ of the real source $S_{t}$ are computed as:

(1) $$S_{t,k}=S_{t}+2\langle p_{k}-S_{t},n_{k}\rangle n_{k}$$

where 〈·〉 is the inner product operator. According to Equation (1), given $n_{k}$ and $p_{k}$, $S_{t,k}$ can be determined by $S_{t}$ using the dimension analysis introduced by Figure 1 in Fu et al. [29]. For example, when k=3 (the east wall), the unit normal $n_{3}=\left(1,0\right)$ and the wall point $p_{3}=\left(L_{x},0\right)$. Supposing a real sound source is located at $S_{t}\left(x,y\right)$, its first-order image sound sources are located at $S_{t,k}\left(x_{t,k},y_{t,k}\right)$ for the kth wall at time t, and then $S_{t,3}\left(x_{t,3},y_{t,3}\right)$ could be $S_{t,3}\left(2L_{x}-x,y\right)$ using Equation (1). Similarly, the other images’ positions could be computed as shown in Table 1.

Denote $r_{e}{}_{t,k}$ as the Euclidean distance between the $S_{t}$ and its $S_{t,k}$ at time t, then:

(2) $$r_{e}{}_{t,k}=∥S_{t}-S_{t,k}∥=\sqrt{{\left(x-x_{t,k}\right)}^{2}+{\left(y-y_{t,k}\right)}^{2}},\;k=1,2,\ldots ,K$$

As the sound propagation speed c is used as a constant here, in the following, we treat distances and propagation times as equivalent. Thus, the first-order echo estimates $d\left(r_{e}{}_{t,k}\right)$ as a delay set for real sound source at time t could be expressed by the difference of $r_{e}{}_{t,k}$ as:

(3) $$d\left(r_{e}{}_{t,k}\right)=\left[r_{e}{}_{t,2}-r_{e}{}_{t,1},r_{e}{}_{t,3}-r_{e}{}_{t,1},\ldots ,r_{e}{}_{t,\hat{k}}-r_{e}{}_{t,1}\right],\;\hat{k}=2,3,\ldots ,K$$

## 4. First-Order Echo Measurements

When the loudspeaker S of the smartphone chirps in an indoor environment, the smartphone microphone M records both the direct path of the sound and its reflections from the walls. Motivated by the robustness of the transfer-function measurement approach based on sequences with better cross-correlation and autocorrelation properties [33], a chirp impulse [16] with similar properties and those more compatible with smartphones [7] was chosen as the emitting signal to simplify the processing of the first-order echo measurements using the generalized cross correlation (GCC) introduced by Knapp et al. [34], which performed well in separating arrivals that were close in time.

### 4.1. Calculation of the Cross-Correlation

The chirp impulse, emitted from S, works between 0≤t≤T with a start frequency $f_{0}$ and an end frequency $f_{1}$, which can be described as:

(4) $$s\left(t\right)=sin\left(2\pi \left(f_{0}+\frac{f_{1}-f_{0}}{2T}t\right)t\right)$$

**Figure 3:**
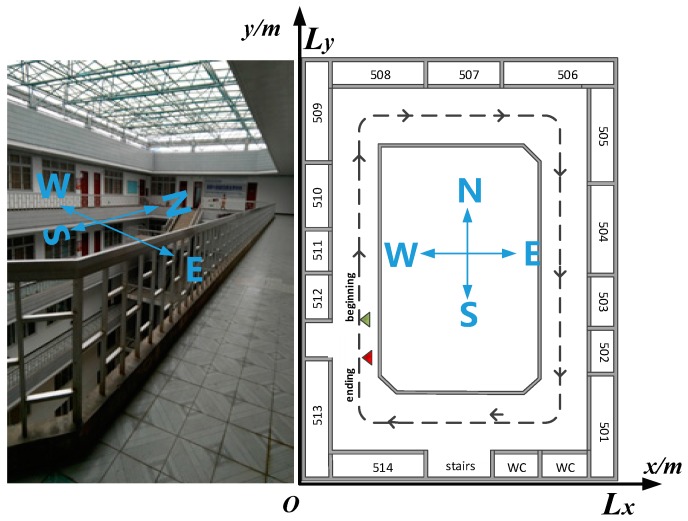
Illustration of the fifth corridor of the Jinji Campus Library in GUET. GUET: Guilin University of Electronic Technology. The dashed lines are the reference walking lines. A small green triangle dot denotes the beginning point and a red one denotes the ending point. The dominant directions are denoted as E: East, S: South, W: West, N: North.

Let the time-domain received signals be r(t), the GCC between the received signals r(t) and the reference signal s(t) is given by the phase transform (PHAT) in time domain:

(5) $$R{\left(\tau \right)}_{s,r}=\frac{1}{2\pi }\int_{-\infty }^{+\infty }\frac{s\left(\omega \right)r^{*}\left(\omega \right)}{\left|s\left(\omega \right)r\left(\omega \right)\right|}e^{j\omega \tau }d\omega$$

where ∗ is the conjugate operator, s(ω) and r(ω) represent the Fourier transforms of the reference signal and the signals received by the microphone of the smartphone, respectively. The GCC-PHAT method has several advantages: first, the correlation between the received signals with a known signal removes uncorrelated noise; second, the implementation of the cross correlation in the frequency domain is more computationally efficient than its implementation in the time domain; third, the PHAT has the ability to decrease the effects of reverberation [35]. In our experiments, since the pedestrian walks along the room’s walls and the dominant directions (east, west, south, north), shown by the reference walking lines in Figure 3, the distance to the four walls are not always equal and the range-resolution is sufficient for path separation. Thus, given the advantage of the chirp’s good correlation characteristics, the GCC-PHAT $R{\left(\tau \right)}_{s,r}$ has the ability to detect the time-of-flights (TOFs), both of the direct path and the reflected path.

### 4.2. Calculation of First-Order Echo Measurements

Given a fixed reflecting surface with a fixed orientation and a sound source point, the expression for the position of the image point can obtained with Equation (1). If also given the boundary values of the room size, this position can be explicitly expressed by Table 1. Thus, according to Equation (2), the distance relationship between the real sound source and its first-order image sound sources can be expressed by taking advantage of the isosceles trapezoid model (ITM), shown in Figure 2b, as:

(6) $$\left\{ \begin{array}{l} r_{m}{}_{t+1,k}=r_{m}{}_{t,1}\pm 2L_{tra}cos\theta ,k=1,3,S_{t+1,x}>S_{t,x}\\ r_{m}{}_{t+1,k}=r_{m}{}_{t,1}\pm 2L_{tra}sin\theta ,k=2,4,S_{t+1,y}>S_{t,y}\\ r_{m}{}_{t+1,k}=r_{m}{}_{t,1}\mp 2L_{tra}cos\theta ,k=1,3,S_{t+1,x}<S_{t,x}\\ r_{m}{}_{t+1,k}=r_{m}{}_{t,1}\mp 2L_{tra}sin\theta ,k=2,4,S_{t+1,y}<S_{t,y} \end{array}\right.$$

where $r_{m}{}_{t,k}$ is the distance between $S_{t,k}$ and $S_{t+1,k}$ for the kth wall at time t. In the aforementioned expression, the dependence on the wall index k is omitted for the sake of brevity; here, k=1 is specifically the west wall. $r_{m}{}_{t+1,k}$ is the distance for the kth wall at time t+1. $S_{t}$ and $S_{t+1}$ with the subscript x or y are the corresponding coordinate values of S at time t and time t+1, respectively.

Since the smartphone is carried by the moving pedestrian, the PDR information—which is regarded as the distance moved (the step length $L_{tra}$), and the movement heading attitude changes (the heading angle θ)—could be solved by the adaptive step length algorithm presented by Shin et al. [30] and a heading correction method similar to the one presented by Deng et al [31]. Denoting WF as the walking frequency when the steps are detected and AV as the acceleration variance, the step length is a linear function of the following measurements:

(7) $$L_{tra}=\left({𝓂}_{wf}\alpha_{wf}\right)WF+\left({𝓂}_{av}\alpha_{av}\right)AV+\left({𝓋}_{wf}+{𝓋}_{av}\right)=\alpha_{Opt}WF+\beta_{Opt}AV+\gamma_{Opt}$$

where ${𝓂}_{wf}$ and ${𝓂}_{av}$ are the measurement errors of WF and AV, respectively, and they are both equal to 0.5, because the measurement errors are minimized. $\alpha_{wf}$ and ${𝓋}_{wf}$, as well as $\alpha_{av}$ and ${𝓋}_{av}$, are the linear fit parameters for WF and AV, respectively. In our experiment, the parameters of $\alpha_{wf}$ and ${𝓋}_{wf}$, as well as $\alpha_{av}$ and ${𝓋}_{av}$, were obtained by averaging the results by recording multiple measurements on the same experimental route. Thus, $\alpha_{Opt},\;\beta_{Opt},\;\mathrm{and}\;\gamma_{Opt}$ are the optimal step length estimation parameters.

As the real paths of the experimenter in this study were along the dominant directions, and during the experiment, the smartphone was always horizontally and statically held in the hand, we simplified the processing of θ by superimposing the *z*-axis angular rate reading $\omega_{z,\kappa }$ from the gyroscope at every step κ. 0≤κ≤K, where K is the total step number:

(8) $$\theta =\overset{K}{\underset{\kappa =1}{\sum }}\omega_{z,\kappa }$$

Similarly, the first-order echo measurements $d\left(r_{m}{}_{t,k}\right)$ as a delay set for S at time t could be

(9) $$d\left(r_{m}{}_{t,k}\right)=\left[r_{m}{}_{t,2}-r_{m}{}_{t,1},r_{m}{}_{t,3}-r_{m}{}_{t,1},\ldots ,r_{m}{}_{t,\hat{k}}-r_{m}{}_{t,1}\right],\;\hat{k}=2,3,\ldots ,K$$

### 4.3. Acoustic Principle-Based Constraints

Since the distance between S and M is very small, i.e.,∥S−M∥→0, the direct sound path from S to M can be described as:

(10) $$\tau_{direct}=arg\underset{\tau }{\max}\left|R{\left(\tau \right)}_{s,r}\right|$$

where |·| is the modulo operation. Similarly, the direct sound paths from $S_{t,k}$ to M can be regarded as the path from $S_{t,k}$ to S, and we denote $\tau_{reflect}$ as a TOF set of these paths as:

(11) $$\tau_{reflect}=\underset{\tau }{\arg}(\left|R{\left(\tau \right)}_{s,r}\right|-max\left|R{\left(\tau \right)}_{s,r}\right|)$$

The first-order echo measurements are provided by solving the unknown top or base values of the isosceles trapezoids that should be the impulse delays in the $R{\left(\tau \right)}_{s,r}$. To reduce the errors of the first-order echo measurements, the acoustic principle-based constraint algorithm is proposed to update the measurements.

#### 4.3.1. Sound Pressure Level Constraint

The Haas effect, also known as the priority effect, reflects the perception of the sound source’s orientation based on the first sound that arrives at the human ear. According to the conclusion of the classic Hass experiment, sounds reflected within 5 to 35 ms after the direct sound can be distinguished when the sound pressure level (SPL) of the reflected sound is greater than 10 dB of the SPL of the direct sound. Thus:

(12) $$SPL_{reflect}-SPL_{direct}\geq 10dB$$

where $SPL_{reflect}$ and $SPL_{direct}$ are the SPLs of the first-order reflections and the direct arrived sound, respectively. Since the sound source is a point source, assume that the image sources are also point sources, so the wavefront is a spherical wave. The expression of spherical acoustic wave attenuation with distance at normal temperature is:

(13) SPL=LW−10lg𝓇−𝓀

where the LW is the sound power level, 𝓇 is the distance between the sound source (the real source or the image source) and the receiver, and 𝓀 is the spacial modifying coefficient. Let $LW_{reflect}$ and $LW_{direct}$ be the sound power level at the real sound source and its first-order image sound source, respectively. Based on the image concepts in AIM, $LW_{reflect}$ = $LW_{direct}$. Then:

(14) $$\begin{array}{cc} SPL_{reflect}- & SPL_{direct}\\ & =LW_{reflect}-20lg{𝓇}_{reflect}-𝓀-\left(LW_{direct}-20lg{𝓇}_{direct}-𝓀\right)\\ & =20lg{𝓇}_{direct}-20lg{𝓇}_{reflect}=20\mathrm{lg}\left(\frac{c\tau_{direct}}{c\tau_{reflect}}\right) \end{array}$$

Thus, $\tau_{reflect}\leq \frac{\tau_{direct}}{\sqrt{10}}$, which means if any first-order reflected sounds within 5 to 35 ms after the direct sound, the $r_{m}{}_{t,k},t\geq 0$ must follow:

(15) $$r_{m}{}_{t,k}\leq \frac{c\cdot \tau_{direct}}{\sqrt{10}}$$

If some of them (the $r_{m}{}_{t,k}$) are outside this range, the known room size $\left[L_{x},L_{y}\right]$ should be used to restrict their values. For example, when the pedestrian walks along the west wall (k=1), $r_{m}{}_{t,1}$ should be the smallest one among all the $r_{m}{}_{t,k}$ values along the west wall phase, $r_{m}{}_{t,1}$ must follow Equation (15); however, the first-order echo delay according to the opposite side (the east wall, k=3) in this phase may be outside the 5 to 35 ms range, then the $r_{m}{}_{t,3}$ value should be restricted by $L_{x}$, i.e., $r_{m}{}_{t,3}=L_{x}-r_{m}{}_{t,1}$. A similar analysis also applies to the $r_{m}{}_{t,2}$ and $r_{m}{}_{t,4}$ with $L_{y}$.

#### 4.3.2. Sound Energy Constraint

Based on the distance relationship between the real sound source and its image sound sources, the propagation delay $\xi_{t,k}$ for any $r_{m}{}_{t,k},t\geq 0$ is:

(16) $$\xi_{t,k}=round\left(\frac{r_{m}{}_{t,k}}{c}\right)$$

where round(·) is the rounding operation. As $\xi_{t,k}$ should be a TOF value in $R{\left(\tau \right)}_{s,r}$, and the computed $\frac{r_{m}{}_{t,k}}{c}$ is not always an integer, a rounding operation is needed. Based on the fact that the energy of the wave is proportional to the square of its amplitude, the pulse amplitude of the cross-correlation function could be used to represent the energy constraint. The sound energy (SE) constraint of the first-order echo impulses according to $\xi_{t,k}$ should be:

(17) $${\left|R{\left(\xi_{t,k}\right)}_{s,r}\right|}^{2}\geq \Delta$$

where Δ is an empirical energy threshold that depends on the room average absorption coefficient. Because the four sides of our experimental environment are glass windows, doors and walls, and the ceiling is mainly glass with steel stent supports (as shown in Figure 3), according to the sound absorption coefficient analysis [36], the sound field is not uniform. Under these conditions, the calculated coefficient will always be smaller than when the sound field is uniform. We calculate the indoor reverberation time according to the Sabine formula, confirming that the room is a high reverberation environment. This may result in the superposition of multiple reflected sounds at the position where the first-order reflected wave occurs. In addition, in large rooms, the sound propagation will experience a long path, when the frequency is above 2 kHz, the air absorption can account for 20–25% of the total sound absorption of the whole space. Therefore, through experimental observation, our empirical energy threshold is set as the following

(18) $$\Delta ={\left(\frac{1}{2K}\overset{K}{\underset{i=1}{\sum }}max\left|R_{i}{\left(\tau_{reflect}\right)}_{s,r}\right|\right)}^{2}$$

#### 4.3.3. Update Algorithm

If the first-order echo measurements $d\left(r_{m}{}_{t,k}\right)$ satisfy the SPL and SE constraints, meaning the PDR is authentic, $d\left(r_{m}{}_{t,k}\right)$ is correct. If not, the PDR is not completely authentic, and $d\left(r_{m}{}_{t,k}\right)$ should be updated by the new values extracted from the constraint range. The above constraint steps are summarized in Algorithm 1.

**Algorithm 1.** Algorithm for Updated First-Order Echo Measurements.Input: $r_{m}{}_{t,k},\;\xi_{t,k},\;\tau_{direct},\;c,\;R{\left(\tau \right)}_{s,r},\;\Delta ,\;\tau_{reflect}$Output: the updated $d\left(r_{m}{}_{t,k}\right)$1: if $r_{m}{}_{t,k}\leq \frac{c\cdot \tau_{direct}}{\sqrt{10}}$ and ${\left|R{\left(\xi_{t,k}\right)}_{s,r}\right|}^{2}\geq \Delta$ then2: $d\left(r_{m}{}_{t,k}\right)\leftarrow d\left(\xi_{t,k}\cdot c\right)$3: otherwise, $\hat{\tau }\leftarrow$ select delays of candidate echos that satisfy both the SPL and the SE constraits4: for all the delay samples of $\xi_{t,k}$ and $\hat{\tau }$ do5: $\underset{t}{\xi_{t,k}\leftarrow argmin}\left(\xi_{t,k}-\hat{\tau }\right)$6: return $\xi_{t,k}$7: end for8: $d\left(r_{m}{}_{t,k}\right)\leftarrow d\left(\xi_{t,k}\cdot c\right)$9: end if

## 5. LMA-WNLS-Based Pedestrian Self-Positioning

Based on the weighted non-linear least squares (WNLS) approach, the cost function is:

(19) $$\varepsilon \left(S_{t}\right)={\left(d\left(r_{m}{}_{t,k}\right)-d\left(r_{e}{}_{t,k}\right)\right)}^{T}*D^{-1}*\left(d\left(r_{m}{}_{t,k}\right)-d\left(r_{e}{}_{t,k}\right)\right)$$

where ${\left(\cdot \right)}^{T}$ is the transpose operation, ${\left(\cdot \right)}^{-1}$ is the inverse operation, and D is the noise covariance matrix. $D=\sigma^{2}I_{K-1}$, where $\sigma^{2}$ is the noise covariance and I is the identity matrix. As estimated distances $r_{e}{}_{t,k}$ and measured distances $r_{m}{}_{t,k}$ are solved by the steps introduced in Section 3 and Section 4, the optimal pedestrian position ${\bar{S}}_{t}$ is:

(20) $${\bar{S}}_{t}=arg\underset{S_{t}}{\min}\varepsilon \left(S_{t}\right)$$

However, the main limitation of the WNLS is that, in order to maintain optimal robustness, its learning rate parameters are usually set to small positives, resulting in a slower convergence rate. Thus, the application of the Levenberg–Marquardt algorithm (LMA) to WNLS could accelerate the convergence while ensuring robustness, and satisfy real-time positioning requirements.

## 6. Experiment

We validated the proposed approach with the data collected from the corridor of the fifth floor of the Jinji Campus Library in GUET, GuiLin, Guangxi Zhuang Autonomous Region, China. The cloister size was $\left[L_{x},\;L_{y}\right]=\left[19,35\right]$. The four sides of the library corridor are doors, glass windows, and walls; the ceiling is mainly glass with steel stent supports; and the floor is covered with ordinary tile. The whole corridor is a rectangular ring.

The data collection tool used in this experiment was a Huawei Rongyao 7 smartphone installed with a chirp application developed by our team and already authorized by China National Intellectual Property Administration, which was used to emit and store the chirp sound signal. The chirp sample frequency was set as $f_{s}=44.1\;\mathrm{kHz}$, the duration was T=0.006 s, the lower frequency was $f_{0}=16\;\mathrm{kHz}$, the upper frequency was $f_{1}=22\;\mathrm{kHz},$ and the emitting interval was 0.3 s. The PDR sample frequency was set as $f_{\mathrm{pdr}}=20\;\mathrm{Hz}$. The empirical energy threshold was set as Δ=0.01.

We had the loudspeaker of the smartphone facing the nearest wall, opened the chirp application, and then walked normally from the starting point (green dot) at [1.5, 9] along the corridor to the end point (red dot) at [1.5, 5]. During data collection, students and staff walked around normally as usual.

### 6.1. Calculation of PDR Information ($L_{tra}$ and θ)

**Figure 4:**
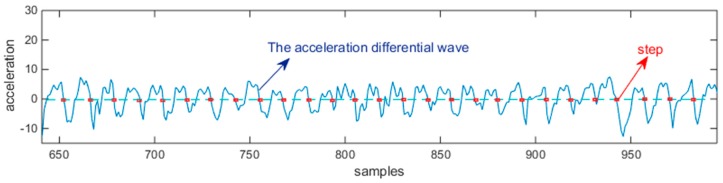
Illustration of a(κ), which is the acceleration pattern of a pedestrian in walking states. The zero crossing points, shown in red rectangles, are the detected steps.

To obtain the adaptive step length $L_{tra}$, the pedestrian acceleration (denoted $a_{NORM}$) was calculated from the norm of the three-axis accelerometer (denoted $∥a_{\kappa }∥$):

(21) $$a_{NORM}=∥a_{\kappa }∥=\sqrt{{\left(a_{x,\kappa }\right)}^{2}+{\left(a_{y,\kappa }\right)}^{2}+{\left(a_{z,\kappa }\right)}^{2}}$$

where $a_{x,\kappa },\;a_{y,\kappa }\;\mathrm{and}\;a_{z,\kappa },\;\kappa =0,\ldots ,K$ are the three-axis accelerometer readings. Then, the sliding window summing technique was used to reduce noise:

(22) $$SWS\left(\kappa \right)=\overset{\kappa }{\underset{t=\kappa -N+1}{\sum }}a_{NORM}\left(t\right)$$

where SWS is the sliding window summing, and the window’s size was set as N=10. Since SWS is affected by walk motion and gravity, the acceleration differential technique was used to obtain the acceleration differential a(κ), as shown in Figure 4:

(23) a(κ)=SWS(κ+N)−SWS(κ)

**Figure 5:**
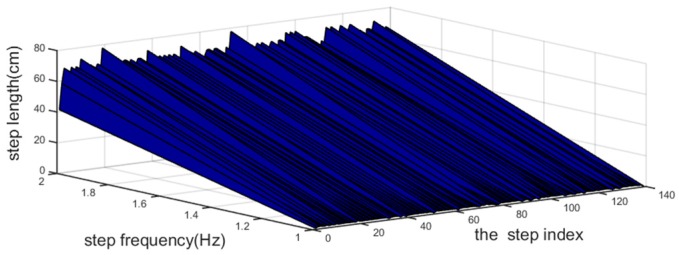
Correlation among $L_{tra}$, K, and step frequency. For each step index κ, the correlation between the $L_{tra}$ and the step frequency satisfied the statistical theory that the step frequency is larger and the step length is longer.

**Figure 6:**
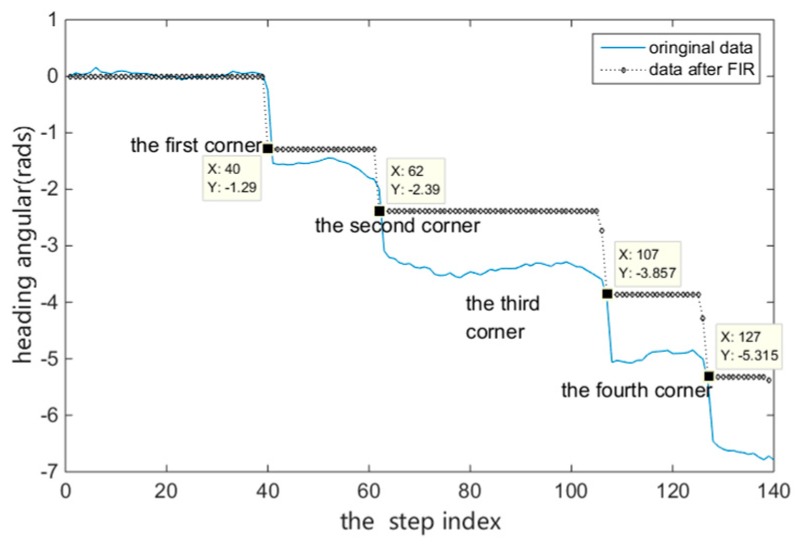
Heading angular θ and the corner step index. The heading angular θ at each κ is shown in a blue full line to clarify the κ of every room corner. FIR technology is applied. The index values of the corridor corners κ are shown in the black numbers.

Using the acceleration measurements, step detection and step length estimation can be accomplished through the walking frequency WF and acceleration variance AV:

(24) $$WF=1/\left(t_{\kappa }-t_{\kappa -1}\right),AV=\left(\overset{M}{\underset{\kappa =1}{\sum }}\left(a\left(\kappa \right)-\bar{a\left(\kappa \right)}\right)\right)/\left(M-1\right)$$

where M and $\bar{a\left(\kappa \right)}$ are the number of samples and the acceleration mean during a step, respectively. Finally, we obtain K=140 from counting the peaks over zero in a(κ) using the find-peaks function. The $L_{tra}$ plot is shown in Figure 5, and the θ plot is shown in Figure 6, which was generated using the method described in Section 4.2.

### 6.2. First-Order Echo Measurements $d\left(r_{m}{}_{t,k}\right)$

**Figure 7:**
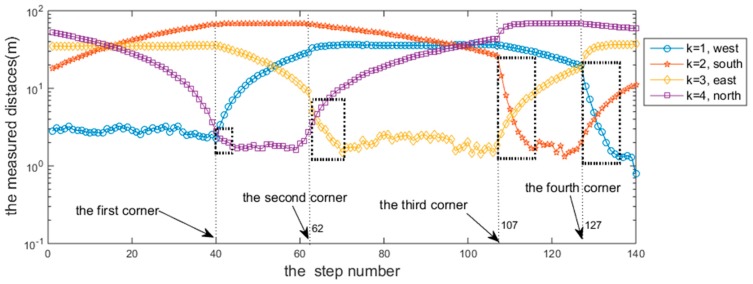
Illustration of the trend diagram of $r_{m}{}_{t,k}$.

When walking along the corridor, the changing trends of distances from the sound source to the four walls were directly reflected in the values of $r_{m}{}_{t,k}$, as shown in Figure 7.

Firstly, from step κ=1 to step κ=40 (the first corner), the user moved from the south to the north. During this phase, the distance from the east wall and the west wall should remain unchanged, the distance from the south wall should be increasingly larger, and the distance to the north wall should be increasingly smaller. Thus, the trajectory trend when 1≤κ≤40 was gentle for k=1 and k=3, increasing for k=2, and decreasing for k=4.

Next, the user moved from the west to the east; that is, from the first corner (κ=40) to the second corner (κ=62). During this phase, the distance from the south wall and the north wall should remain unchanged, the distance from the west wall should increase, and the distance to the east wall should decrease. Thus, the trajectory trend when 40≤κ≤62 is gentle for k=2 and k=4, increasing for k=1, and decreasing for k=3.

Similarly, the trends of the distance changes for other sections were the same as the changes in the actual distances.

However, the change parts marked with the black dotted rectangles at every corner point in Figure 7, which should be the smooth transition curves, become sudden sharp declines. After repeating the measurements, we think that the reason for this change is the remaining accumulated errors of the heading angle θ due to the assumption that the experimenters in this study walked strictly along the dominant directions. In fact, the randomness of a person’s walking causes their direction of travel to deviate from the dominant direction, and this error is also eventually reflected in the trajectory of the position tracking.

**Figure 8:**
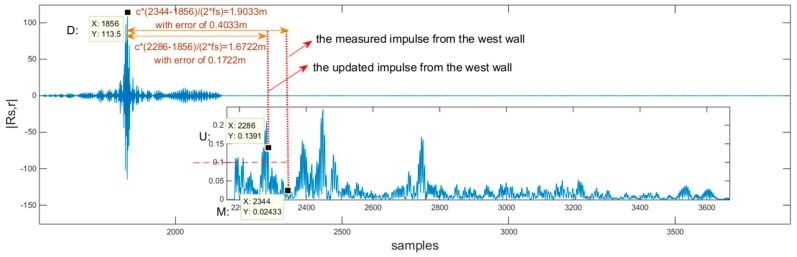
$R{\left(\tau \right)}_{s,r}$ when κ=140,k=1. The $R{\left(\tau \right)}_{s,r}$ wave is generated when the step index of the pedestrian is κ=140, the impulses marked in red dot lines are the reflections from the west wall.

To further explain the $r_{m}{}_{t,k}$ extracted from the cross-correlation $R{\left(\tau \right)}_{s,r}$, Figure 8 shows the $r_{m}{}_{t,k}$ in the $R{\left(\tau \right)}_{s,r}$ when κ=140, k=1.

1. The direct path impulse was found at the peak with maximum value $\left|R{\left(\tau \right)}_{s,r}\right|$, which is marked by D:(X:1856,Y:113.5);
2. We subtracted $R{\left(\tau \right)}_{s,s}$ from $R{\left(\tau \right)}_{s,r}$ to eliminate the waveform sidelobe effect and amplify the reverberation parts, as shown in the lower right corner of Figure 8, to find the real first-order echo impulses;
3. Since κ=140>127, we deduced that the pedestrian has passed the fourth corner and should be on the west side of the corridor, so the peak marked with M:(X:2344,Y:0.02433) generated by the $r_{m}{}_{t,k}$ at this moment was taken as the first-order reflection from the west wall (i.e., the closest wall); however, ${\left|Y\right|}^{2}\approx 0.0006\ll \Delta =0.01$, the measured result did not meet the SE constraint, and so should be updated;
4. With the measured $r_{m}{}_{t,3}$ and the constraint of $r_{m}{}_{t,1}\approx L_{x}-r_{m}{}_{t,3}$, based on the proposed algorithm, the first-order reflection peak related to $r_{m}{}_{t,1}$ was updated with the value marked with U:(X:2286,Y:0.1391), which had a smaller distance error than the one before the update, thereby reducing the error of the position; the other first-order reflection peaks were gradually found, and updated.

### 6.3. Self-Positioning Trajectory Comparison

**Figure 9:**
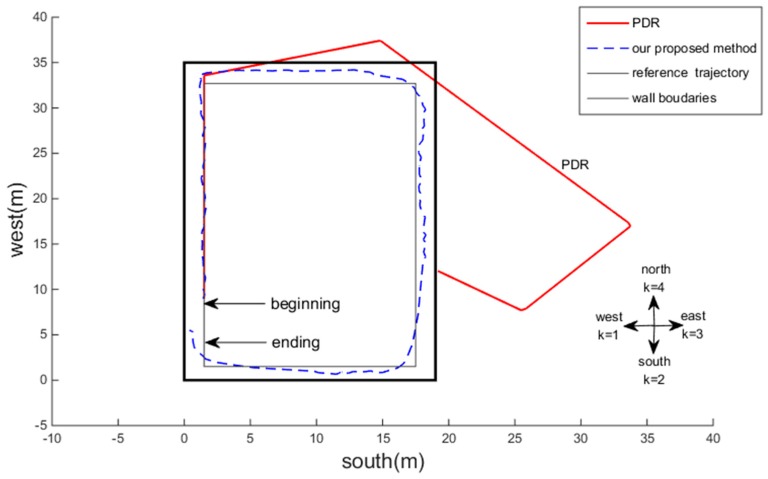
Trajectory comparison of the pedestrian dead reckoning (PDR) method and our proposed method.

To highlight the advantages of our proposed continuous sound source self-positioning solution, we used two strategies: PDR and our proposed system. The compared results are shown in Figure 9. The following can be seen from the figure: (1) The output of the PDR trajectory (the red line) is continuous and has a similar shape to the reference trajectory (the gray line), but as time increased and the number of pedestrian steps increased, accumulative errors occurred in the accelerometer and gyroscope, resulting in positioning failure. (2) The proposed system output (the blue short line) is closer to the reference trajectory, because it accounts for the acoustic constraints to confirm the required K=4 dimension distances between the sound source and its image sources, increasing the accuracy of the positioning result, determined by the starting point to the first corner point, and the trajectory is closer to the reference trajectory.

For the same reason as mentioned above, due to the inherent defect of the angle estimation method (an angular cumulative error that cannot be totally eliminated), there were some fluctuations in the corner areas in the tracking trajectory, which is consistent with the change parts marked with the black dotted rectangles in Figure 7, but overall, it was closer to the reference trajectory.

### 6.4. Error Analysis

**Figure 10:**
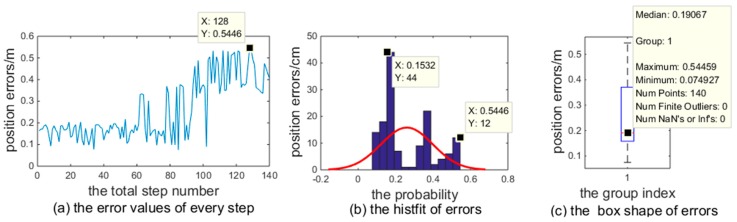
Analysis of the proposed system errors. (**a**) Error values of every step. Here, the step number K=140; (**b**) error probability distribution histogram produced by the MATLAB histfit function based on the data of (**a**); and (**c**) result of the MATLAB box function based on the data of (**a**), describing the distribution of the collected data and visualizing the normalities and abnormalities of the data.

The errors presented in Figure 8 are illustrated in Figure 10 with the following outcomes:

(1) When κ increased, the positioning error increased, as shown in Figure 10a. The error could be as great as 0.5446 m, but the probability was rather low (w.r.t.1/140=0.71%);
(2) As shown in Figure 10b, the errors of each step were centralized by the histfit function, the probability of error below 0.12 m was 54.46%, and the probability of the error exceeding 0.44 m did not exceed 15.32%;
(3) The box figure (Figure 10c) details the median, maximum, and minimum of the proposed system errors. This result proves that the proposed system is reliable.

## 7. Conclusions

We proposed a sensor-rich smartphone-based indoor pedestrian self-positioning system for continuous position acquisition based on image acoustic source impulse. Along with the processing, an acoustic principle-based constraint algorithm was proposed to update the first-order echo measurements generated from the PDR and ITM methods, increasing the reliability of the final positioning results compared to the PDR method. Additionally, the LMA-WNLS model was adopted to reduce the computational complexity of the continuous self-positioning process, thereby increasing time efficiency. Despite this, we noticed some limitations of this system. For example, the used smartphone must have an application that can emit and receive chirp sounds because it is impossible for ordinary smartphones to play chirp sound signals. The arbitrariness of pedestrian motion during walking is limited. If the actual trajectory of walking deviates from the dominant direction, heading angle errors are produced, resulting in positioning error.

Related future work will mainly focus on the data processing of the heading angle and the separation of the close echo arrivals, to further improve the positioning accuracy and fully port this complete system to a smartphone application.

## Figures and Tables

**Table 1:** Suppose a real sound source is located at $S_{t}\left(x,y\right)$: its first-order image sound sources are located at $S_{t,k}\left(x_{t,k},\;y_{t,k}\right)$ for different k at any time t. The corresponding coordinates and reflection orders are shown below.

Coordinate	−1st Order	Real Source	1st Order
*x*	$S_{t,1}\left(-x,y\right)$	$S_{t}\left(x,y\right)$	$S_{t,3}\left(2L_{x}-x,y\right)$
*y*	$S_{t,2}\left(x,-y\right)$	$S_{t}\left(x,y\right)$	$S_{t,4}\left(x,2L_{y}-y\right)$
